# Congenital renal anomalies detected in adulthood

**DOI:** 10.2349/biij.8.1.e7

**Published:** 2012-01-01

**Authors:** M Muttarak, T Sriburi

**Affiliations:** Department of Radiology, Chiang Mai University, Chiang Mai, Thailand.

**Keywords:** renal anomalies, horseshoe kidney, crossed renal ectopia, malrotation, ultrasound

## Abstract

**Objective:**

To document the types of congenital renal anomalies detected in adulthood, the clinical presentation and complications of these renal anomalies, and the most useful imaging modality in detecting a renal anomaly.

**Materials and methods:**

This study was approved by the institutional review board and informed consent was waived. Between January 2007 and January 2011, the clinical data and imaging studies of 28 patients older than 18 years diagnosed with renal anomaly at the authors’ institution were retrospectively reviewed. Renal anomalies in this study included only those with abnormality in position and in form.

**Results:**

Of these 28 patients, 22 underwent imaging studies and their results constituted the material of this study. Of the 22 patients, 14 had horseshoe kidneys (HSK), four had crossed renal ectopia and four had malrotation. Sixteen patients were men and six were women. The patients ranged in age from 19 to 74 years (mean age 51.1 years). Clinical presentations were abdominal pain (13), fever (13), haematuria (4), palpable mass (2), asymptomatic (2), polyuria (1) dysuria (1), blurred vision (1), and headache with weakness of left extremities (1). Imaging studies included abdominal radiograph (15), intravenous pyelography (IVP) (8), retrograde pyelography (RP) (4), ultrasonography (US) (7), and computed tomography (CT) (9). Associated complications included urinary tract stones (17), urinary tract infection (16), hydronephrosis (12), and tumours (2). Abdominal radiograph suggested renal anomalies in nine out of 15 studies. IVP, RP, US and CT suggested anomalies in all patients who had these studies performed. However, CT was the best imaging modality to evaluate anatomy, function and complications of patients with renal anomalies.

**Conclusion:**

HSK was the most common renal anomaly, with abdominal pain and fever being the most common presentations. UTI and stones were the most common complications. IVP, RP, US and CT can be used to diagnose renal anomalies but CT is the best imaging modality to evaluate renal anatomy, function and its complications.

## INTRODUCTION

Congenital abnormalities of the kidney and urinary tract occur in approximately 3.3–11.1% of the population and they account for about 50% of all congenital abnormalities [1]. Congenital abnormalities of the kidney are a leading cause of end-stage renal disease (ESRD) in children and also cause subsequent renal problems in adulthood because many renal anomalies predispose the patient to obstruction, which leads to stone formation, infection, hypertension, and renal failure. Imaging plays an important role in early diagnosis and proper management. Ultrasonography (US) is a non-invasive modality and has a major impact on evaluating renal anomalies either prenatally or postnatally [2]. However, it may be difficult to diagnose a small damaged kidney or dysplastic kidney with US. Other imaging studies such as computed tomography (CT) and occasionally, magnetic resonance imaging (MRI), are used because they can generate three-dimensional images and are not disrupted by bowel gas. CT and MRI are useful to confirm the presence or absence of an anatomic abnormality or mass lesions in the kidneys that are congenitally of unusual shape and in which the parenchyma may be difficult to assess adequately by US, e.g, crossed, fused ectopia and dysplastic kidneys. Renal function can be evaluated by intravenous pyelography (IVP) as well as CT [2–6]. Familiarity towards the imaging features of renal anomalies and their complications will provide early diagnosis and proper management and will decrease the incidence of ESRD. The purpose of this study is to document the types of congenital renal anomalies detected in adulthood, the clinical presentation and complications of these renal anomalies, and the type of imaging modality that is most useful in detecting a renal anomaly.

## MATERIALS AND METHOD

The local institutional review board approved this study and informed consent was waived. Between January 2007 and January 2011, the hospital registry was searched to identify patients aged over 18 years with diagnosis of renal anomaly. Renal anomaly in this study included only those with abnormality in position and in form. There were 28 patients diagnosed with renal anomaly. Their clinical data and imaging studies were retrospectively reviewed by one senior radiologist and one 3^rd^ year resident. Imaging studies included abdominal radiograph, IVP, retrograde pyelography (RP), US and CT. Agreement on the imaging findings was achieved by consensus. US was obtained using a variety of commercially available scanners using 3.5 MHz transducers (HDI 5000, Advanced Technology Laboratories, Bothell, WA, USA, Siemens, Sequoia, Acuson, CA, USA, and Toshiba Aplio XG, Japan). CT was performed using multidetector CT (MDCT) scanner (Aquilion 16, Toshiba, Tochigi-Ken, Japan and Somatom Definition, Siemens, Germany). Approximately 100–150 ml of non-ionic contrast material containing 320–370 mg/ml of iodine was administered intravenously at the rate of 4 ml/second.

## RESULTS

There were 28 patients diagnosed as having renal anomaly. Of these 28 patients, 22 underwent imaging studies and their results constituted the material of this study. Of the 22 patients, 14 had horseshoe kidneys (HSK), four had crossed renal ectopia and four had malrotation. The patients ranged in age from 19 to 74 years (mean age 51.1 years). Clinical presentations were abdominal pain (13), fever (13), haematuria (4), palpable mass (2), asymptomatic (2), polyuria (1) dysuria (1), blurred vision (1) and headache with weakness of left extremities (1). Complications resulting from each anomaly is shown in table 1. Imaging studies included abdominal radiograph in 15 patients, IVP in eight, retrograde pyelography (RP) in four, US in seven, and CT in nine patients. None of them had genital anomalies.

**Table 1:** Tube current used for pediatric head routine CT in three hospitals.

**Complications**	**Anomalies**		
	**Horseshoe kidney** **N = 14**	**Crossed ectopia** **N = 4**	**Malrotation** **N = 4**
Stone	11	3	3
UTI	10	4	2
Hydronephrosis	6	3	3
Tumour	2	0	0

**Figure 1:**
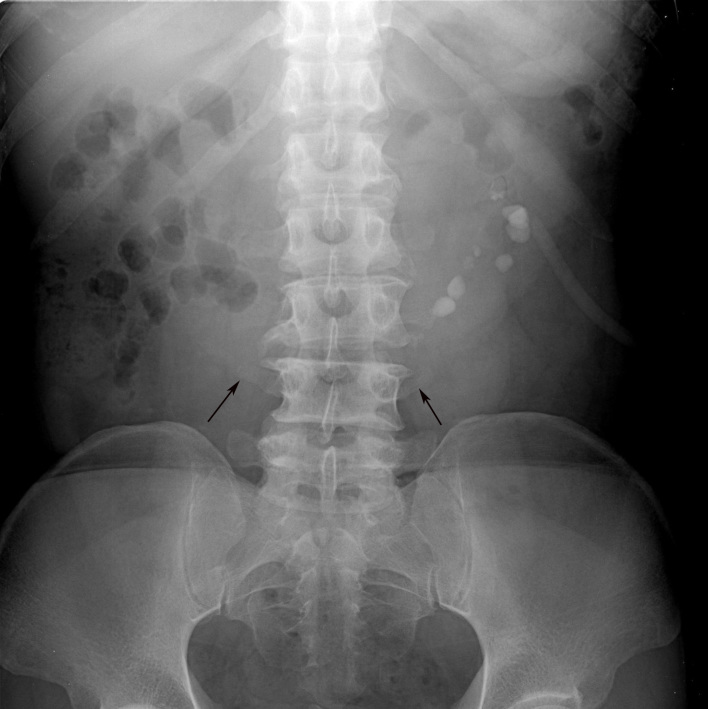
Abdominal radiograph in a 50-year-old man who presented with abdominal pain shows medial deviation of the lower poles of both kidneys and the visible isthmus (arrows). There are multiple stones in the left kidney and stones in the lower pole locate medially while stones in the upper pole locate laterally.

**Figure 2:**
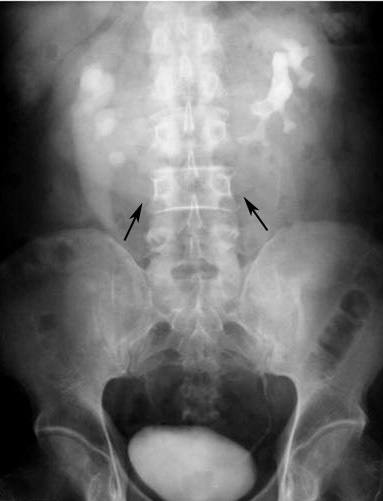
IVP shows a characteristic appearance of HSK in a 48-year-old man who presented with abdominal pain. The two kidneys lie vertically on either side of the midline, and are joined at their lower poles by an opacified parenchymal isthmus (arrows). The right renal pelvis is directed laterally and the left renal pelvis is directed anteriorly. Bilateral hydronephrosis is also seen.

**Figure 3:**
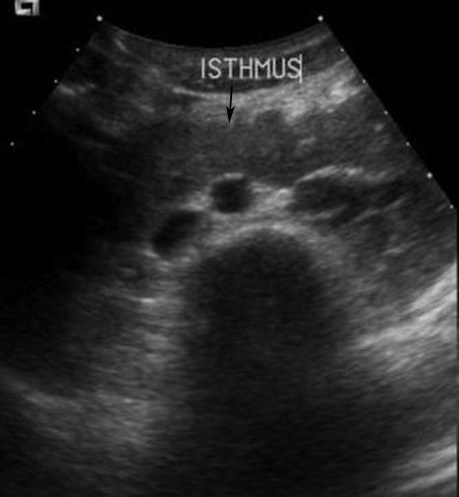
Transverse US image of a 19-year-old man who presented with haematuria shows a hypoechoic isthmus (arrow) lying anterior to the aorta and vertebral body.

**Figure 4:**
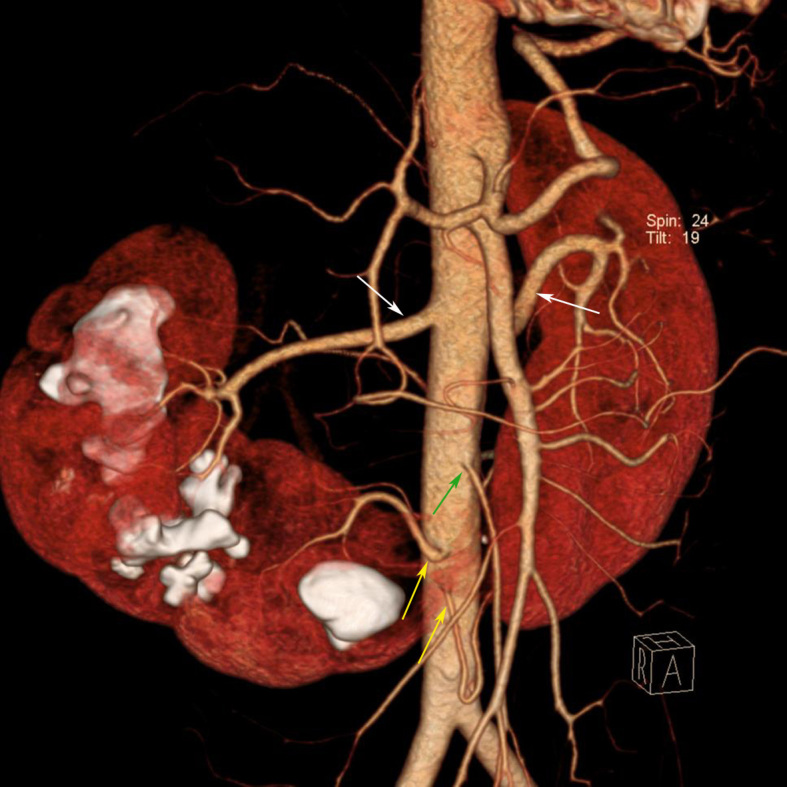
A 40-year-old man presented with abdominal pain and diagnosis of horseshoe kidney was made by IVP. MDCT was performed for surgical planning. Coronal volume rendering MDCT image shows various blood supply to the horseshoe kidney. Right and left renal arteries (white arrows) supply the upper and middle pole of each kidney, two aortic branches (yellow arrows) supply the lower pole of both kidneys and the isthmus. The isthmus is just below the IMA (green arrow) origin. Multiple renal stones are also seen in the right kidney.

**Figure 5:**
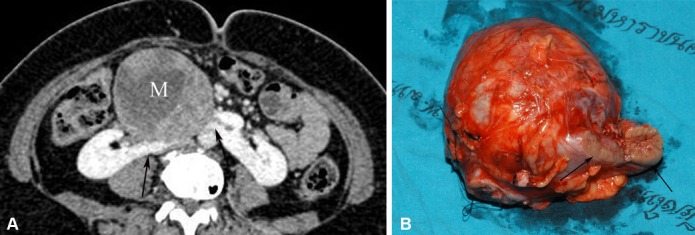
Neuroendocrine renal carcinoma occurring in a HSK. (A) Axial-enhanced MDCT image in a patient presenting with a palpable abdominal mass shows a large heterogeneous enhancing mass (M) originating from an enhancing isthmus (arrow) that lies anterior to the aorta. (B) Photograph of the gross specimen after isthmectomy and tumour resection shows a large circumscribed tumour and isthmus (arrows).

Of the 14 patients with HSK, 12 were men and two were women. All of them had abnormal rotation of the kidneys and fusion at the lower poles as a U-shape. For two patients who had a palpable mass, one was proven to have neuroendocrine renal cell carcinoma and the other one was suspected to have transitional cell carcinoma but the patient refused surgery. Imaging findings of HSK from each modality (Figures 1–5) are shown in table 2.

**Table 2:** Summary of imaging findings from each imaging modality in 14 patients with horseshoe kidney.

	**Number of patients by imaging study**			
	**Abdominal radiograph**	**IVP**	**US**	**CT**
Findings	9	4	2	8
Visible renal axis	5	4	2	8
- Vertical	2	0	0	0
- Medial deviated lower pole	3	4	2	8
Visible isthmus	4	3	2	8
- Fibrous	Cannot evaluate	Cannot evaluate	0	2
- Parenchyma			2	6
Arterial supply to kidney	Cannot evaluate	Cannot evaluate	Cannot evaluate	
- Renal artery				8
- Aortic branch				7
- Common iliac artery				1
Isthmus				
- Aortic branch				5
- Common iliac artery				4
Stones	9	4	1	7
- Medial position	8	4	Cannot evaluate	7
Hydronephrosis	Cannot evaluate	3	1	3
Abnormal renal function	Cannot evaluate	1	Cannot evaluate	1
Mass	0	0	0	2

**Figure 6:**
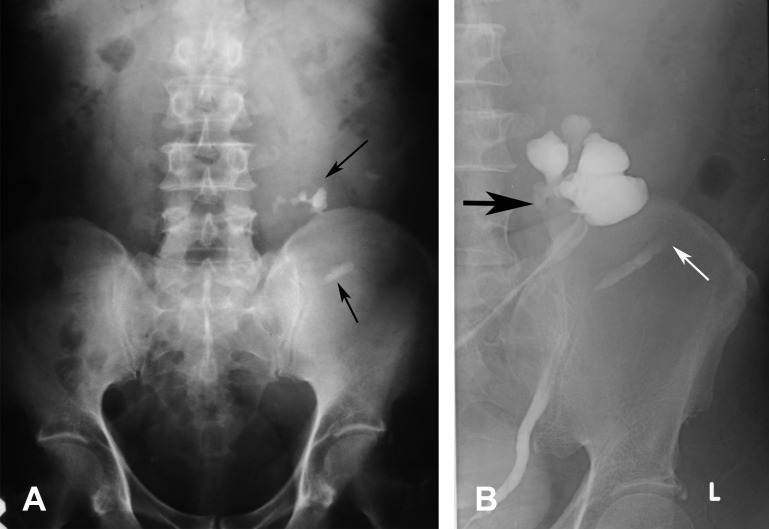
A 56-year-old man presented with fever and blurred vision. He was found to have diabetes mellitus, hypertension, and high creatinine level. (A) Abdominal radiograph shows multiple stones (arrows) in the left side of the abdomen which are lower than the normal renal position and nonvisualised right renal outline in the right renal fossa. US abdomen (not shown) showed no kidney in the right side and left hydronephrosis. (B) RP shows right crossed kidney with nonrotation and hydronephrosis (black arrow), and moderate hydronephrosis. The left ureter is laterally deviated with nonvisualised left kidney due to obstructed stone which is seen as a filling defect (white arrow).

**Figure 7:**
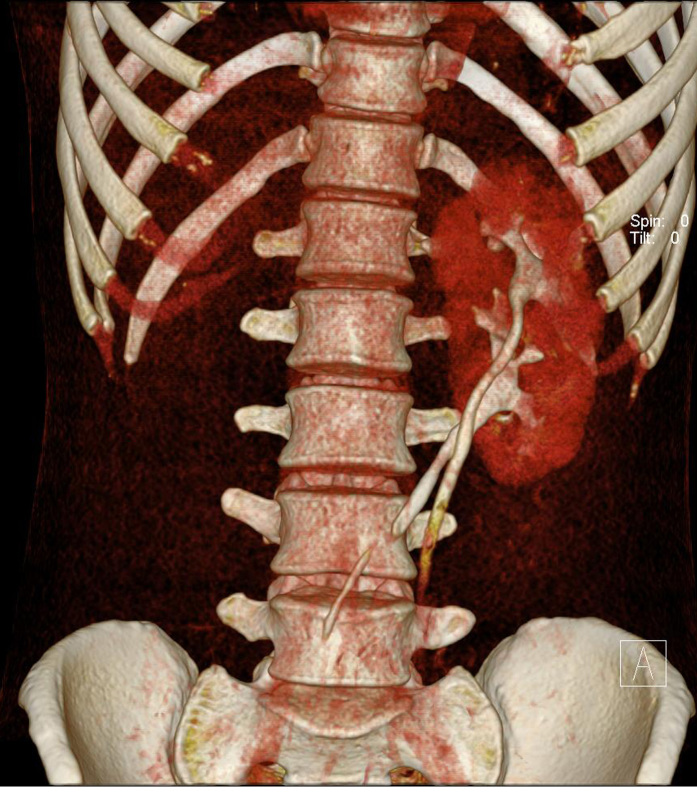
A 28-year-old man presented with headache and weakness of the left extremities. He was found to have basal ganglia haemorrhage and hypertension. US abdomen (not shown) showed no kidney in the right renal fossa. MDCT was done for surgical planning. Coronal volume rendering MDCT image shows fused kidneys with two separate pelvocalyceal systems and ureters. The right ureter crosses midline to the left side and the right kidney fuses to the lower pole of the left kidney.

Of the four patients with crossed renal ectopia, three were men and one was a woman. Of these four patients, three had crossed kidney in the opposite paravertebral region and one had crossed kidney in the opposite side of the pelvic cavity. All of them had unilateral crossed kidney and one was fused. One patient had crossed left kidney to the right and three had crossed right kidney to the left. Imaging findings of crossed renal ectopia from each modality (Figures 6 and 7) are shown in table 3.

**Table 3:** Summary of imaging findings from each imaging modality in 4 patients with crossed renal ectopia.

	**Number of patients by imaging study**				
	**Abdominal radiograph**	**IVP**	**RP**	**US**	**CT**
Findings	2	1	2	4	1
Visible renal outline	1	1	2	4	1
Crossed kidney	Not seen	Left ureter crossed to the right but left kidney was not visualised	2 crossed right kidneys to left paravertebral region	1 crossed left kidney to right pelvis. 1 crossed right kidney to left paravertebral region	1crossed right kidney to left paravertebral region below left kidney	Visible renal axis						- Vertical	1	0	2	Cannot evaluate	1	- Normal	0	0			0	Rotation	Cannot evaluate			Cannot evaluate		- Incomplete		0	2		1	Fusion	Cannot evaluate	Cannot evaluate	Cannot evaluate	Cannot evaluate	1	Arterial supply to kidney	Cannot evaluate	Cannot evaluate	Cannot evaluate	Cannot evaluate		- Renal artery						- Aortic branch					1						1	Stones	2	1	2	3	0	- low position	1	0	1	0		Hydronephrosis	Cannot evaluate	1	2	2	0	Abnormal renal function	Cannot evaluate	1	Cannot evaluate	Cannot evaluate	0

**Figure 8:**
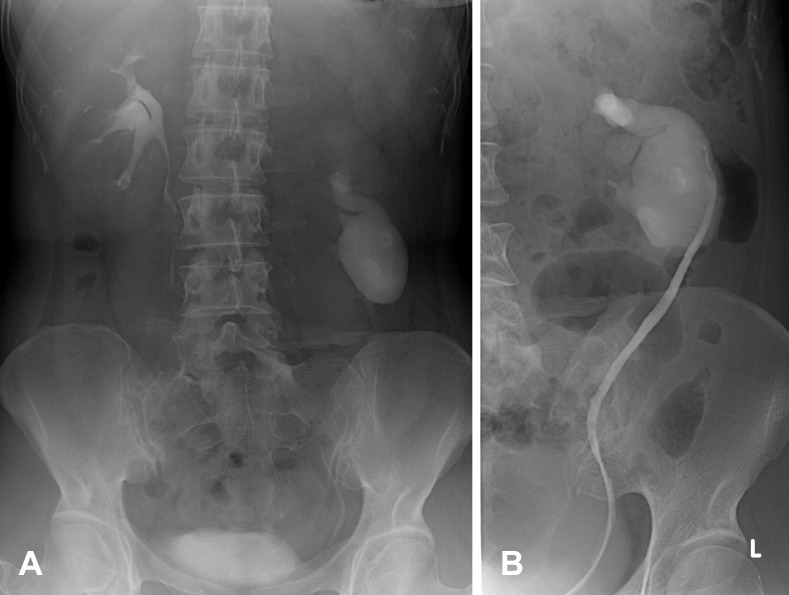
A 51-year-old man presented with a palpable left abdominal mass. (A) IVP shows reverse rotation of the left kidney with marked hydronephrosis. The left kidney locates lower than normal. (B) RP of the same patient shows lateral pointing of the left pelvocalyceal system and its ureter without ureteric obstruction.

All of the four patients with malrotation had unilateral malrotation, and three were women while one was a man. Three of these four patients were reverse rotation and one was nonrotation. Imaging findings of malrotation from each modality (Figure 8) are shown in table 4. The position of the malrotated kidney was lower than normal in three patients and normal in one.

**Table 4:** Summary of imaging findings from each imaging modality in 4 patients with malrotation.

	**Number of patients by imaging study**			
	**Abdominal radiograph**	**IVP**	**RP**	**US**
Findings	4	3	2	1
Visible renal axis	3	3		
- Vertical	1	2	2	Cannot evaluate
- Normal	2	1		
Rotation				
- Nonrotation	Cannot evaluate	1	0	1
- Reverse rotation		2	2	0
Stones	2	2	2	1
- Low position	1	2	2	1
Hydronephrosis	Cannot evaluate	2	2	0
Abnormal renal function	Cannot evaluate	0	Cannot evaluate	Cannot evaluate

## DISCUSSION

The kidneys develop during the 4^th^ week of gestation by the union of the ureteric bud with the metanephric mass of intermediate mesoderm at the level of the first or second sacral segment. The kidneys initially lie close to each other with the hila directed anteriorly in the pelvis. As the abdomen and pelvis grow, the kidneys gradually ascend to the lumbar region and move further apart. During ascent, the kidneys rotate medially almost 90 degrees so that the hila are eventually directed anteromedially. By the 9^th^ week of gestation, they attain their adult position adjacent to the adrenal gland [2]. Anomalies of the kidneys can be classified into anomalies in number, in size, in position, in form (fusion), and in structure [7]. This study included only those with anomalies in position and in form. This study found 14 patients with HSK, four with crossed renal ectopia and four with malrotation.

HSK is the most common renal fusion anomaly, with an incidence of approximately 0.25% in the general population, occurring more frequent in men (2.3:1) than women [8]. In this study, 12 were men and two were women. HSK may occur as an isolated entity but approximately one-third is associated with a wide variety of genitourinary and non-genitourinary anomalies [7]. No associated anomaly was seen in this study. Fusion of the kidneys occurs when they are still in the pelvis but HSK may be found anywhere along the normal embryologic ascent of the kidneys. However, a HSK usually lies in a lower position than the normal kidneys because the isthmus does not permit ascent beyond the inferior mesenteric artery (IMA). Most cases of HSK are fused at the lower poles by an isthmus of renal parenchyma or fibrous tissue that crosses the midline of the body. The isthmus usually lies anterior to the great vessels at the level of the third to fifth lumbar vertebra, just below the origin of the IMA from the aorta. Rarely, the isthmus lies posterior to these vessels or may run between them. All the 12 patients in this study had fusion at the lower poles.

Axis and rotation of the fused kidneys are abnormal. Each kidney lies more vertically than normal, the lower poles deviate medially than the upper poles. The renal pelvises and ureters locate more anteriorly or laterally than normal. The ureters usually cross anterior to the isthmus as they descend into the bladder. In this study, associated malrotation can be identified by CT in all eight patients and by IVP in four patients. Blood supply to HSK is variable and may originate from the aorta, the iliac arteries and the IMA. Because of the abnormal course of the ureters, they are prone to obstruction. Hence, 20%–60% of patients with HSK have stones [9, 10]. However, approximately one-third of patients with HSK are asymptomatic and a HSK is diagnosed as an incidental finding [11]. When symptoms are present, they are usually related to obstruction, stones or infection. Imaging plays an important role in the evaluation of these patients because symptoms are often non-specific. Associated renal neoplasms such as Wilm’s tumours in children, renal carcinoid tumour, and transitional cell carcinoma have been reported to be increased in patients with HSK [7]. Though renal cell carcinoma (RCC) is the most common associated malignancy in patients with HSK, there is controversy regarding the increased incidence of RCC in patients with HSK [4, 12–14]. In this study, 11 patients had renal stones, 10 had UTI, six had hydronephrosis, two had renal masses, and two were asymptomatic. Of the two patients with renal mass, one was proven to have neuroendocrine renal cell carcinoma and the other one was suspected to have transitional cell carcinoma but the patient refused surgery. One of the two asymptomatic patients was incidentally diagnosed as having HSK during abdominal CT staging for his lymphoma. The other one had microscopic haematuria from his urinalysis check up and was referred for imaging study.

Diagnosis of HSK may be obtained on abdominal radiograph as observation of the renal outline. Four kinds of abnormal renal outlines [8] suggestive of HSK are 1) lower than normal, 2) too close to the spine, 3) vertical long axis, and 4) visible isthmus. In this study, HSK was suggested in five out of nine abdominal radiographs. Although IVP is decreasing in use throughout the world, it is still used in the authors’ institute to evaluate renal outlines and function. The isthmus composed of renal parenchyma will opacify together with the rest of the kidney during nephrographic phase of the IVP study. However, IVP cannot reliably differentiate a fibrous isthmus from a parenchymal isthmus. If surgery is planned, CT is often required.

In this study, HSK was diagnosed on IVP in all four patients who performed this study but the isthmus was visible in three. On US, the isthmus is visualised as a midline hypoechoic lesion joining the two lower poles of the kidneys. If the isthmus is small or composed of fibrous tissue or hidden by bowel gas, it may be difficult to identify by US (4, 15). The axis of HSK may be difficult to detect by US. Therefore familiarity towards the US features of a HSK will improve accuracy in diagnosis. US was performed in only two patients in this study and diagnosis of HSK could be made in both.

MDCT is a comprehensive imaging modality to detect HSK. It is able to depict the anatomy of the whole urinary tract and to evaluate its complications such as stone, hydronephrosis, infection or tumors. Contrast enhancement MDCT can evaluate the function of the kidney, differentiate functioning isthmus from fibrous tissue and provide information about the blood supply of the fused kidney, which is helpful for surgical planning. Because of the distorted anatomy in patient with renal anomaly, CT is useful for treatment planning such as CT-guided drainage and CT-guided puncture for percutaneous nephrolithotripsy (PCNL) [15]. The main limitations of MDCT are high radiation dose and increased risk of allergy to iodinated contrast media. In this study, MDCT was performed in eight patients and it was the best imaging modality to delineate anatomy, function and complications of the HSK.

MRI and scintigraphy using technetium-labelled radionuclides (^99m^Tc-dimercaptosuccinic acid or glucoheptonate) can also be used for diagnosis of HSK but these two modalities were not performed in this study.

Crossed renal ectopia refers to a kidney that locates on the contralateral side of its ureteric orifice. This anomaly occurs predominantly in males. In this study, this anomaly was more common in men than in women (3:1). The crossed kidney is ectopic, usually lies below and with the upper pole of the crossed kidney fused to the lower pole of the orthotopic kidney. Crossed ectopia without fusion is rare. The ureter of the crossed kidney crosses the midline and enters the bladder on the opposite site. Crossover of the left kidney to the right side is the most common form of this anomaly but one patient was found with left kidney crossed to the right and three had right kidney crossed to the left and one was fused. Variants of this anomaly have been described but the two kidneys are usually vertically-oriented and the ectopic kidney is often malrotated [5, 7]. Blood supply to the ectopic kidneys usually arises from the vessels in the vicinity, and they are often supplied by multiple vessels. Patients with crossed renal ectopia are usually asymptomatic and are often discovered incidentally. However, patients may present with signs and symptoms of urinary tract obstruction, UTI or a palpable mass because the crossed ectopic kidney is likely to be complicated by ureteropelvic obstruction. All the four patients with crossed renal ectopia in this study had UTI and three had stones with hydronephrosis.

Diagnosis of crossed renal ectopia may be suggested from abdominal radiograph if one renal outline is not visualised and the opposite renal outline is enlarged or when stones are seen at unusual positions. IVP shows the absence of a kidney in its normal position and the two kidneys on the same side of the abdomen vertically oriented one above the other. The limitation of IVP is that it cannot be performed in patients with poor renal function. In these circumstances, RP or US can provide better information. US can determine if the kidney is in its normal renal fossa. The presence of the two kidneys on one side and the absence of a kidney in the contralateral side are suggestive of crossed ectopia. In this study, abdominal radiograph could suggest crossed ectopia in one patient because of the absence of one renal outline. RP was performed in two patients and diagnosis of crossed ectopia could be made. US was performed in all four patients and absence of the kidney in one side was found in all patients. However, the crossed kidney was found in the other side in only two patients. CT can accurately demonstrate the kidney and its abnormal relationship very clearly [2, 7, 16–18]. In this study, CT was performed in only one patient and diagnosis of crossed ectopia with fusion was accurately made.

During the ascent of the kidney, it normally rotates to anteromedially. Malrotation occurs if it fails to rotate or overrotate. This anomaly can be unilateral or bilateral and is usually associated with abnormal position or fusion of the kidney. This form of anomaly is classified into nonrotation, incomplete rotation, reverse rotation, transverse rotation, and excessive rotation. The most common rotational anomalies are nonrotation and incomplete rotation [2, 7]. Patients with renal malrotation have no specific complications, but affected kidneys are prone to the same diseases that affect a normal kidney [7]. However, three of the four patients in this study had associated renal stones with hydronephrosis and two had UTI. It is difficult to diagnose malrotation with abdominal radiograph. IVP can better delineate renal outline, axis and its function. US may suggest malrotation if the renal pelvis is directly anterior to the calyces and the renal parenchyma. Again, CT can show the type of malrotation very well. In this study, this anomaly was not seen on abdominal radiograph but was diagnosed by IVP in three and by RP in two patients. US was performed in only one patient and diagnosis could be made by demonstration of the anteriorly directed renal pelvis.

## CONCLUSION

HSK was the most common abnormality seen in this study, followed by malrotation and crossed renal ectopia. Abdominal pain and fever were the most common presentations. Most of the patients in this study had stones or UTI, and two patients with HSK had renal tumours. Imaging study plays an important role to diagnose these anomalies and their complications. Abdominal radiograph may suggest these renal abnormalities but it is not specific. US plays an important role in diagnosis of HSK and crossed ectopia but it is difficult to diagnose malrotation. CT is the best imaging method to evaluate renal anomalies, function and their complications. Familiarity of imaging features of renal anomalies and their complications will provide early diagnosis and proper management.
